# Aging‐induced aberrant RAGE/PPARα axis promotes hepatic steatosis via dysfunctional mitochondrial β oxidation

**DOI:** 10.1111/acel.13238

**Published:** 2020-09-16

**Authors:** Jian Wan, Xiangsong Wu, Hanbei Chen, Xinyi Xia, Xi Song, Song Chen, Xinyuan Lu, Jie Jin, Qing Su, Dongsheng Cai, Bin Liu, Bo Li

**Affiliations:** ^1^ Department of Emergency and Critical Care Medicine Shanghai Pudong New Area People's Hospital Shanghai University of Medicine and Health Sciences Shanghai China; ^2^ Department of General Surgery XinHua Hospital Shanghai Jiao Tong University School of Medicine Shanghai China; ^3^ Department of Endocrinology XinHua Hospital Shanghai Jiao Tong University School of Medicine Shanghai China; ^4^ Department of Endocrinology and Metabolism Shanghai Jiao Tong University Affiliated Sixth People's Hospital Shanghai China; ^5^ Department of Molecular Pharmacology Diabetes Research Center Institute of Aging Albert Einstein College of Medicine Bronx NY USA; ^6^ Hubei Key Laboratory for Kidney Disease Pathogenesis and Intervention Hubei Polytechnic University School of Medicine Huangshi China

**Keywords:** aging, hepatic steatosis, mitochondria, PPARα, RAGE

## Abstract

Non‐alcoholic fatty liver disease (NAFLD), characterized by an increase in hepatic triglyceride (TG) content, is the most common liver disease worldwide. Aging has been shown to increase susceptibility to NAFLD; however, the underlying molecular mechanism remains poorly understood. In the present study, we examined hepatic TG content and gene expression profiles in body weight‐matched young (3 months old), middle‐aged (10 months old), and old (20 months old) C57BL/6 mice and found that TGs were markedly accumulated while mitochondrial β‐oxidation‐related genes, including PPARα, were downregulated in the liver of old mice. In addition, advanced glycation end product receptor (RAGE), a key regulator of glucose metabolism, was upregulated in the old mice. Mechanistically, suppression of RAGE upregulated PPARα and its downstream target genes, which in turn led to reduced TG retention. Finally, we found that hepatic RAGE expression was increased in aging patients, a finding that correlated with decreased PPARα levels. Taken together, our findings demonstrate that the upregulation of RAGE may play a critical role in aging‐associated liver steatosis.

## Graphical abstract

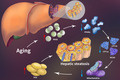

## 1 INTRODUCTION

Non‐alcoholic fatty liver disease (NAFLD), characterized by aberrant triglyceride (TG) accumulation in the liver, has become one of the most common liver diseases worldwide and affects as many as one‐third of all adults in developed countries (Cohen, Horton, & Hobbs, 2011). Growing evidence suggests that aging is an important risk factor in the development of NAFLD (Cree et al., 2004; Fan et al., 2005; Gong, Tas, Yakar, & Muzumdar, 2017). Other studies showed that elderly participants were markedly insulin‐resistant compared with young controls because of the increased fat accumulation in liver tissues of the older individuals (Flannery, Dufour, Rabol, Shulman, & Petersen, 2012; Petersen et al., 2003). However, the molecular mechanism underlying the initiation and/or progression of NAFLD in the elderly population remains largely unknown.

The hallmark of NAFLD is TG accumulation in the liver, which is derived from an imbalance in TG synthesis and clearance. Intracellular hepatic free fatty acid (FFA) catabolism is regulated mainly via the mitochondrial β‐oxidation systems by which FFAs are broken down. Impaired mitochondrial FFA β‐oxidation systems are considered to among the major mechanisms contributing to liver steatosis (Friedman, Neuschwander‐Tetri, Rinella, & Sanyal, 2018; Mansouri, Gattolliat, & Asselah, 2018). Indeed, clinical studies have shown that patients with non‐alcoholic steatohepatitis have mitochondrial dysfunction and impaired β‐oxidation in the liver (Pessayre & Fromenty, 2005; Sanyal et al., 2001). At the molecular level, hepatic mitochondrial β‐oxidation is mainly regulated by PPARα, which drives the expression of target genes involved in mitochondrial β‐oxidation, such as carnitine palmitoyltransferase 1a (*CPT1a*), carnitine palmitoyltransferase 1b (*CPT1b*), and medium‐chain acyl‐CoA dehydrogenase (*MCAD*). Liver PPARα expression has been implicated as crucial in the development of hepatic steatosis (Montagner et al., 2016; Zhao et al., 2018). On the other hand, activation of PPARα and mitochondrial β‐oxidation ameliorates liver steatosis in preclinical models (Li et al., 2014; Pawlak, Lefebvre, & Staels, 2015), indicating a new potential therapeutic area.

Initially, advanced glycation end product receptor (RAGE) has been considered a central regulator of neural system (Cho, Xie, & Cai, 2018; Osgood, Miller, Messier, Gonzalez, & Silverberg, 2017) and has been suggested to play an important role in aging‐related arterial diseases (Senatus & Schmidt, 2017; Yamagishi & Matsui, 2018). RAGE also showed important roles in liver damage as well as in insulin resistance (Chandrashekaran et al., 2017; Song et al., 2014). Advanced glycation end products (AGEs), one of the most important ligands of RAGE, are well known for their roles in hyperglycemia and insulin resistance (Bidasee et al., 2004; Bierhaus & Nawroth, 2009). Besides, our recent study indicated that AGE‐RAGE signaling could promote the proliferation of colorectal and liver cancer cells (Chen et al., 2014). However, whether RAGE plays a role in the development of aging‐associated hepatosteatosis has not been investigated.

In the present study, we hypothesized that RAGE dysfunction might be involved in the development of hepatosteatosis during aging. Our data support the causal role of the RAGE/PPARα pathway in hepatosteatosis and provide novel insights into aging‐induced fatty liver.

## 2 RESULTS

### 2.1 Increased hepatic triglyceride content and RAGE expression in aging mice

**FIGURE 1:**
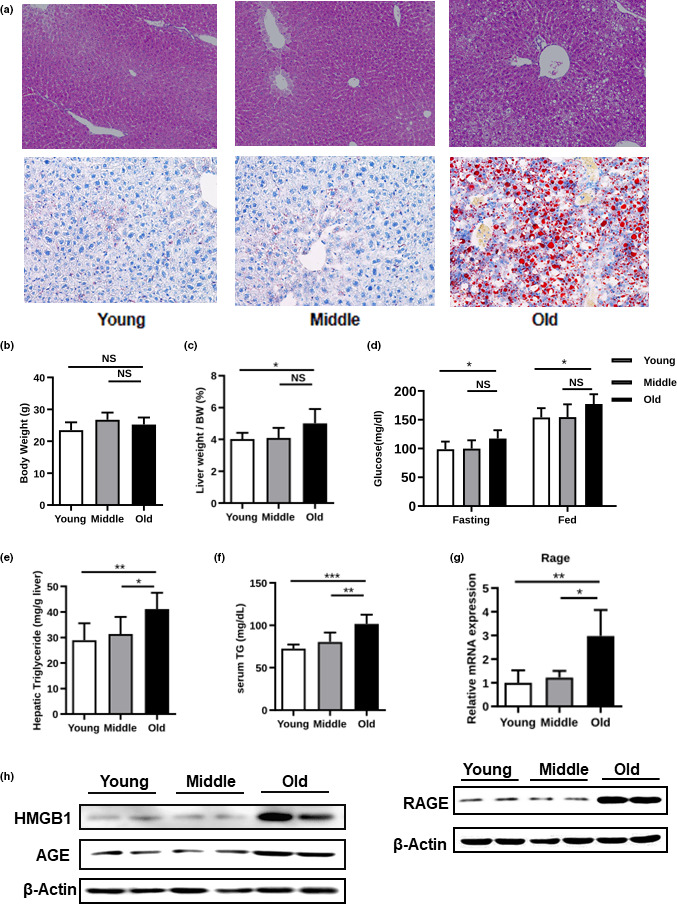
NAFLD‐associated phenotypes in young, middle‐aged, and old C57BL/6 mice. (a) H&E‐ and oil red O (ORO)‐stained liver sections, (b) body weight, (c) liver‐to‐body weight ratio, (d) blood glucose, (e) hepatic triglycerides (TGs), (f) serum TG level, (g) real‐time PCR analysis of gene expression of *RAGE*, and (h) expression levels of HMGB1, AGE, and RAGE, as determined by Western blotting analysis

To evaluate hepatic TG metabolism in aging mice, we performed H&E and Oil Red O (ORO) staining of the liver sections from C57BL/6 mice aged 3 months (young), 10 months (middle‐aged), and 20 months (old) (Figure 1a). To rule out the effect of obesity on lipid deposition, we selected body weight‐matched mice (Figure 1b). As shown in Figure 1c,d, the liver‐to‐body weight ratio and blood glucose were higher in the old mice. Hepatic TG accumulation and hypertriglyceridemia were also observed in the old mice (Figure 1e,f). To explain the potential mechanism for hepatic TG accumulation in aging mice, we examined the expression of RAGE, which was markedly upregulated in the old mice (Figure 1g). In addition, the expression of HMGB1 and AGE, two critical ligands of RAGE, was also increased in the livers of the old mice (Figure 1h), suggesting that the RAGE pathway is activated in aging mice.

### 2.2 RAGE regulates hepatic steatosis in aging

**FIGURE 2:**
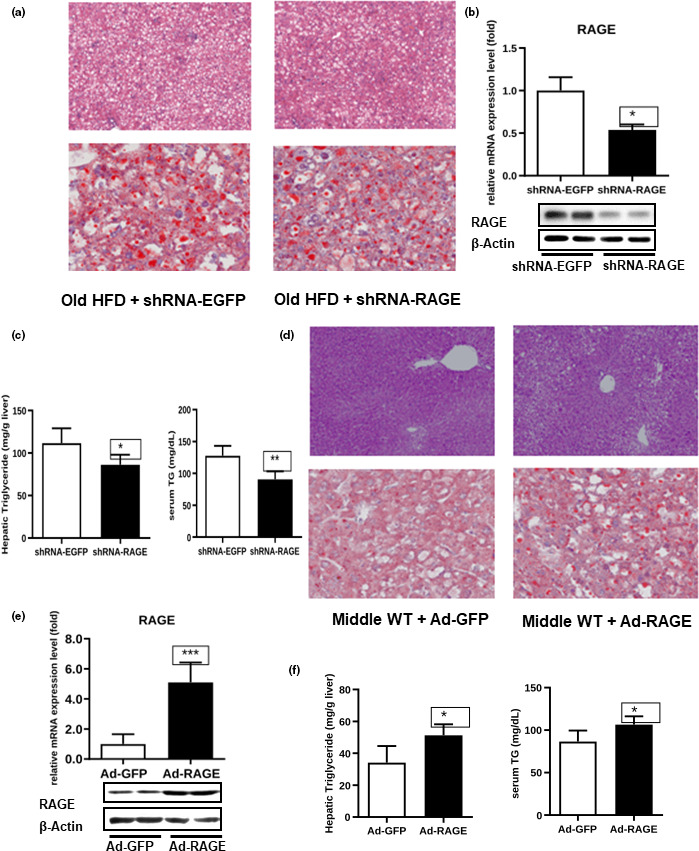
Effect of shRNA‐RAGE and Ad‐RAGE on TG levels in mice. Old mice with HFD were injected with shRNA‐EGFP or shRNA‐RAGE virus, and RAGE knockdown efficiency was determined by real‐time PCR (b). In addition, assays for H&E and oil red O (ORO) staining of liver sections (a), Western blotting (b), and hepatic and serum TG levels (c) were performed. Middle‐aged wild‐type mice were injected with Ad‐GFP or Ad‐RAGE virus, and RAGE overexpression efficiency was determined by real‐time PCR (e). Assays for H&E‐ and oil red O‐stained liver sections (d), Western blotting (e), and hepatic and serum TG levels (f) were performed

To study the role of RAGE in hepatic steatosis in aging, we injected adenoviruses expressing RAGE shRNA or a control scrambled adenovirus (shRNA‐EGFP) into aged C57BL/6 mice with high‐fat diet (HFD). As expected, we detected significantly lower hepatic lipid deposition and RAGE levels in the mice infected with RAGE shRNA than in the mice infected with the control virus (Figure 2a,b). Functionally, RAGE knockdown dramatically decreased hepatic TG accumulation and serum TG levels (Figure 2c). A similar phenomenon was also observed when we injected adenoviruses expressing RAGE shRNA or shRNA‐EGFP into aged wild‐type C57BL/6 mice (Figure S1A–C). Importantly, RAGE knockdown also improved hyperglycemia, and insulin resistance in aged C57BL/6 mice with steatosis, as revealed by glucose and insulin tolerance tests (Figure S2A,B). Due to a close association between hepatosteatosis and inflammation, RAGE knockdown inhibited macrophage infiltration and expression of pro‐inflammatory cytokines, including Tnfa, Il1b, Il12b, Nos2, Ccl2, Ccl5, and Adgre1 (Figure S2C,D).

To further identify the role of RAGE in hepatic steatosis during aging, we tried another approach: We injected Ad‐RAGE adenovirus and its respective control adenovirus (Ad‐GFP) into middle‐aged C57BL/6J WT mice. As expected, we detected significantly higher hepatic lipid deposition and RAGE levels in mice infected with the Ad‐RAGE virus than in control mice (Figure 2d,e). In agreement with these results, RAGE overexpression dramatically increased hepatic TG accumulation and serum TG levels (Figure 2f). We also observed a glucose intolerance and reduced insulin sensitivity in mice overexpressing RAGE (Figure S2E,F). Moreover, liver‐specific overexpression of RAGE caused significant macrophage infiltration and expression of pro‐inflammatory cytokines in the liver of middle age mice (Figure S2G,H).

### 2.3 RAGE regulates hepatic PPARα expression

**FIGURE 3:**
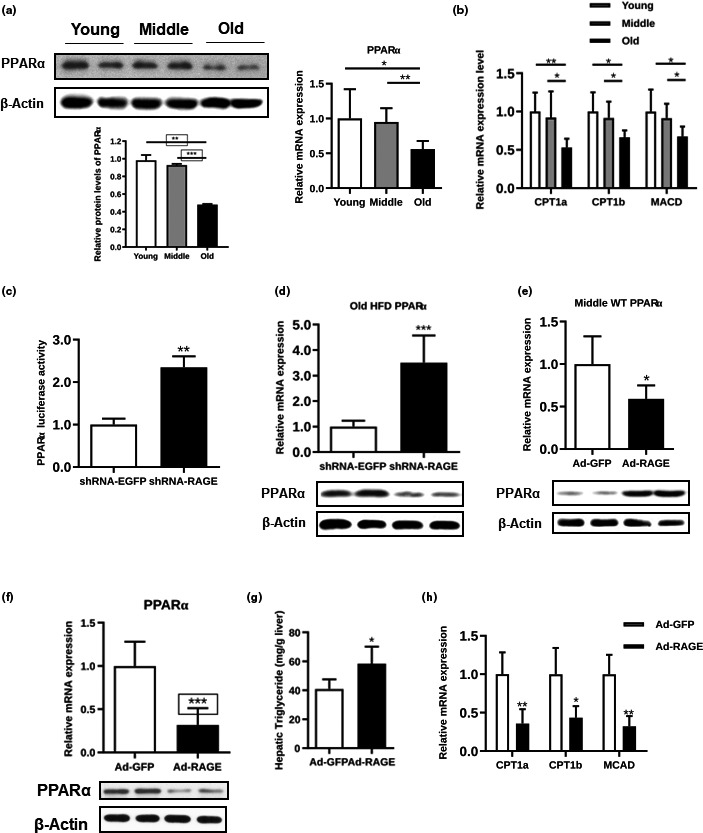
AGE and RAGE expression plays a role in hepatic fatty acid β‐oxidation. The expression levels of PPARα in mice of different ages were examined by Western blotting and real‐time PCR (a), and the expression levels of *CPT1a*, *CPT1b*, and *MCAD* were measured with real‐time PCR (b). (c) PPARα luciferase activity of the HEK293T cells transfected with Ad‐GFP or Ad‐RAGE. (d) PPARα efficiency was determined by real‐time PCR and Western blot analysis in the old HFD mice infected with shRNA‐EGFP or shRNA‐RAGE virus. (e) PPARα expression was determined by real‐time PCR and Western blot analysis in the middle‐aged WT mice infected with Ad‐GFP or Ad‐RAGE. (f–h) PPARα expression in the WT mice as determined by real‐time PCR and Western blot analysis (f), hepatic TG (g), and *CPT1a*, *CPT1b*, and *MCAD* efficiency as determined by real‐time PCR (h) in the primary hepatocytes transfected with Ad‐GFP or Ad‐RAGE

We sought to investigate the molecular basis for RAGE in hepatic TG accumulation. PPARα (encoded by *Ppara*), a master regulator of mitochondrial FFA β‐oxidation (Lu et al., 2014), was particularly reduced in the aging mice (Figure 3a). Consistently, its downstream genes, including *CPT1a*, *CPT1b*, and *MCAD*, were also reduced in the old mice (Figure 3b). Moreover, we detected significantly higher *CPT1a*, *CPT1b*, and *MCAD* expression in old mice infected with RAGE shRNA than in old mice infected with the control virus (Figure S1E), suggesting that RAGE may negatively regulate PPARα expression. To test this hypothesis, we cotransfected the PPARα reporter construct with RAGE shRNA virus or its negative control into HEK293T cells. We found that the PPARα activity was significantly increased in the group transfected with the RAGE shRNA virus (Figure 3c). In addition, PPARα expression was increased in the old HFD mice infected with RAGE shRNA virus (Figure 3d), the increment of PPARα expression was also observed in the old wild‐type mice infected with RAGE shRNA virus (Figure S1D), demonstrating that PPARα expression was reversely correlated with RAGE expression level. In agreement, PPARα expression was decreased in the middle‐aged mice injected with Ad‐RAGE (Figure 3e), and overexpression of RAGE in the young C57 wild‐type mice induced hepatic TG accumulation and decreased the expression of PPARα and its target genes (Figure 3f–h). However, the body weights, fat pad masses, and the expression levels of fibrosis‐related genes were not affected (Figure S4A‐C). Treatment of mice with Ad‐RAGE and Ad‐GFP with or without fenofibrate (Feno), a PPARα agonist, hepatic TG accumulation, and serum TG levels were dramatically decreased by Feno (Figure S4D,E), PPARα target genes were also increased accordingly (Figure S4F). These data support that the hypothesis that hepatic PPARα activity is suppressed by RAGE.

### 2.4 RAGE/PPARα regulates hepatic mitochondrial β‐oxidation of FFAs in vitro

**FIGURE 4:**
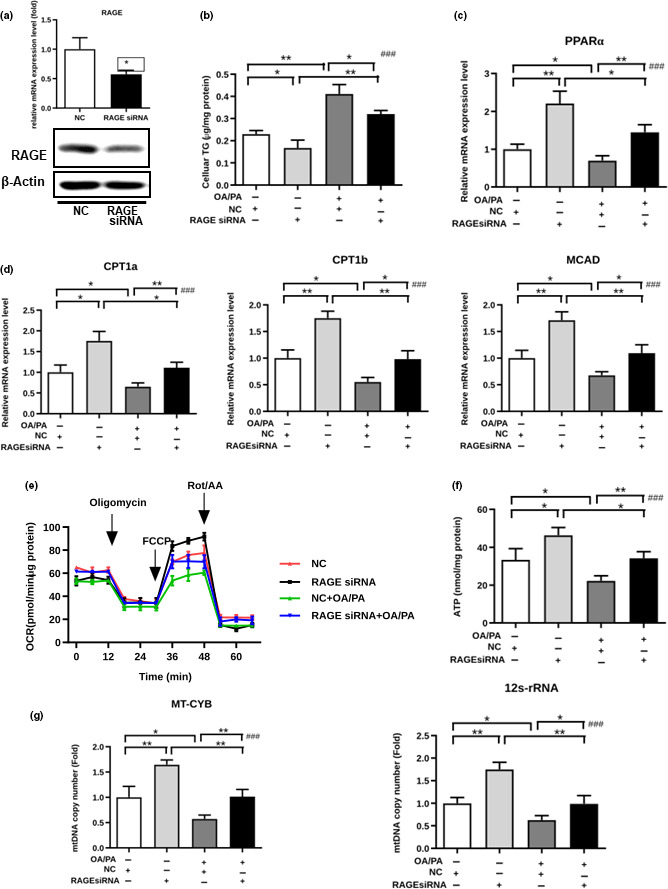
RAGE regulates hepatic FFA mitochondrial β‐oxidation in vitro. (a) PPARα expression was determined by real‐time PCR and Western blot analysis in the primary hepatocytes transfected with RAGE siRNA or control siRNA. (b–g) After the primary hepatocytes were transfected with RAGE siRNA or NC siRNA and stimulated with OA/PA or the BSA control, the cellular TGs (b), expression levels of PPARα (c), *CPT1a*, *CPT1b*, and *MCAD* (d), intact cellular oxygen consumption rate in real time (e), cellular ATP levels (f), and copy numbers of MT‐CYB and 12S rRNA (g) were examined

To determine the role of the RAGE/PPARα axis in hepatic FFA metabolism, we first transfected primary hepatocytes with RAGE siRNA and validated the knockdown efficiency (Figure 4a). Then, the cells were exposed to oleate acid/palmitate acid (OA/PA), two abundant FFAs in the serum of obese mice (Escande et al., 2010), or a BSA control. As a result, less lipid accumulation was detected in the RAGE siRNA‐transfected cells than was detected in the control cells (Figure 4b), indicating that RAGE silencing in hepatocytes led to reduced lipid accumulation under both basal and OA/PA‐stimulated conditions. Consistently, gene expression analysis also showed that the expression of PPARα and its downstream target genes, *CPT1a*, *CPT1b*, and *MCAD*, was increased by suppression of RAGE (Figure 4C,D).

Next, we analyzed the oxygen consumption rate (OCR) of primary hepatocytes to establish the relationship between RAGE and mitochondrial respiration. Knockdown of RAGE increased mitochondrial respiration under both basal and OA/PA exposure conditions (Figure 4e). In addition, ATP production was markedly decreased by OA/PA treatment, suggesting that normal mitochondrial function plays an important role in FFA metabolism. We found that silencing RAGE led to higher ATP content under both basal and OA/PA exposure conditions (Figure 4f). The copy number of two mtDNA genes, *cytochrome b* (*MT*‐*CYB*) and *12S rRNA*, which are related to mitochondrial biogenesis (Hänfling et al., 2016), also increased when RAGE was silenced (Figure 4g). These results indicate that RAGE downregulation increased both basal and maximal mitochondrial respiration as well as cellular ATP production.

**FIGURE 5:**
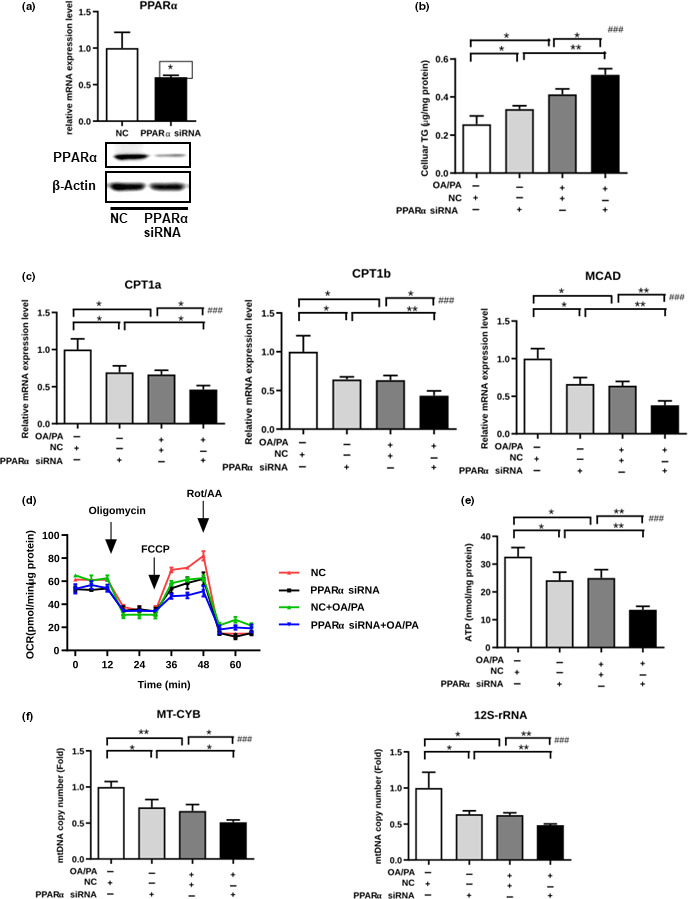
PPARα regulates hepatic mitochondrial β‐oxidation of FFAs in vitro (a) PPARα expression as determined by real‐time PCR and Western blotting analysis in primary hepatocytes transfected with PPARα siRNA or non‐silencing control siRNA. (b–f) After primary hepatocytes were transfected with PPARα siRNA or NC siRNA and stimulated with OA/PA or the BSA control, the cellular TG (b), expression levels of *CPT1a*, *CPT1b*, and *MCAD* (c), intact cellular oxygen consumption rate in real time (d), cellular ATP levels (e), and copy numbers of MT‐CYB and 12S rRNA (f) were examined

Consistently, silencing PPARα in primary hepatocytes by siRNA (Figure 5a) also resulted in greater lipid accumulation (Figure 5b) and decreased mitochondrial FFA β‐oxidation‐related gene expression, including that of *CPT1a*, *CPT1b*, and *MCAD* (Figure 5c). Furthermore, PPARα silencing decreased the OCR, ATP content, and copy number of *MT*‐*CYB* and 12S rRNA (Figure 5d–f). These results indicate that PPARα downregulation decreased both basal and maximal mitochondrial respiration as well as cellular ATP production.

Because AGE and HMGB1 are ligands of RAGE (Bangert et al., 2016; Bierhaus & Nawroth, 2009), we used aminoguanidine (AG), an AGE inhibitor, to block AGE and HMGB1 siRNA to inhibit HMGB1 (Figure S3A,B). We found that OA/PA‐induced lipid accumulation was alleviated by AG and HMGB1 siRNA (Figure S3C,D), demonstrating that both AGE and HMGB1 play important roles in RAGE‐mediated hepatic steatosis.

### 2.5 Aberrant RAGE/PPARα axis in aging individuals with hepatic steatosis

**FIGURE 6:**
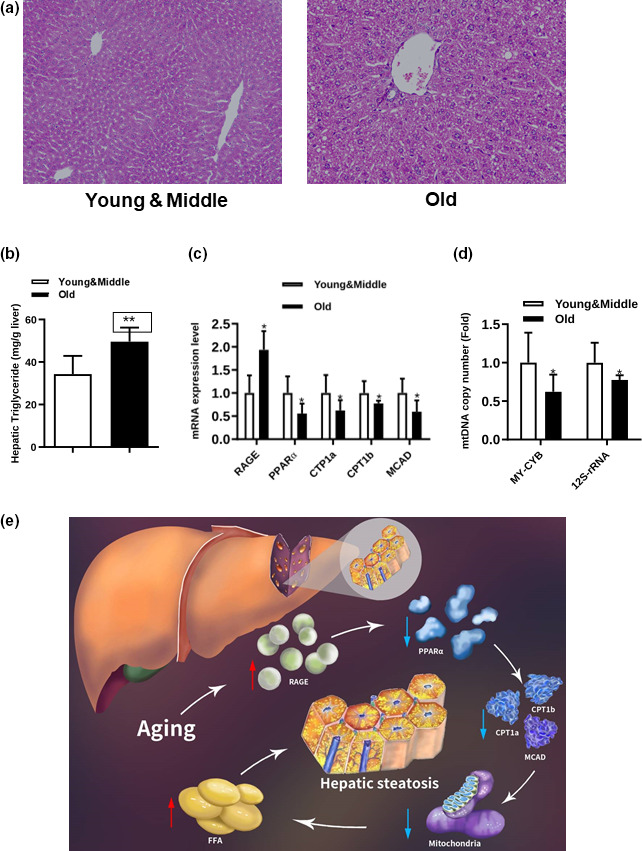
RAGE/PPARα pathway in hepatic steatosis patients. (a–d) H&E‐stained liver sections (a), hepatic TG levels (b), expression levels of RAGE, PPARα, CPT1a, CPT1b, and *MCAD* (c) and copy numbers of MT‐CYB and 12S rRNA (d) of NAFLD patients separated into two groups. (e) Schematic model: We propose that aging induces RAGE upregulation, which inhibits PPARα signaling and suppresses mitochondria‐related fatty acid β‐oxidation, eventually leading to excessive FFA production and then hepatic steatosis

Finally, we explored the clinical relevance of the RAGE/PPARα axis to hepatic steatosis in aging individuals. We observed that the old individuals appeared to have more lipid accumulation in liver sections based on H&E staining (Figure 6a). Interestingly, hepatic TG content and RAGE expression were markedly elevated, while PPARα and its target genes were downregulated in these individuals (Figure 6b,c). In addition, the copy number of *MT*‐*CYB* and *12S rRNA* was reduced in these individuals (Figure 6d). These findings strongly support a correlation between the RAGE/PPARα axis and hepatic steatosis in aging individuals.

## 3 DISCUSSION

In the current study, we showed that hepatic TG was markedly accumulated in aging mice compared with body weight‐matched young mice. At the molecular level, we found that RAGE was upregulated in the aging mice. In agreement with these results, knockdown of RAGE led to increased expression of PPARα and downstream β‐oxidation‐related genes, with improved mitochondrial function in vitro, while knockdown of PPARα had the opposite effects. Importantly, we provided evidence of increased hepatic RAGE levels in aging patients, which correlated with decreased PPARα levels and β‐oxidation. To our knowledge, this study is the first to illustrate a novel biologic function of RAGE in regulating aging‐associated hepatic steatosis.

One of the main characteristics of an aging liver is hepatic steatosis, which is also the earliest step in NAFLD. Age‐associated hepatic steatosis is controlled by several pathways. Although previous studies indicated that endoplasmic reticulum stress, NAD^+^ deficiency, and ghrelin hormone deletion may be important causes of NAFLD pathogenesis in aging (Guillory et al., 2018; Xiong et al., 2014; Zhou et al., 2016), the molecular mechanism for aging‐induced fatty liver remains poorly understood. It has been reported that alterations of the chromatin structure occur in livers of old mice, suggesting that age‐related epigenetic changes might be involved in hepatic steatosis. The transcriptional factor C/EBPα is phosphorylated at Ser193 by CDK4 in livers of old mice and can recruit p300 and C/EBPβ to form a tripartite p300‐C/EBPα/β complex that activated promoters of five genes that drive triglyceride synthesis. Indeed, knockdown of p300 or inhibition of CDK4 in old mice inhibits hepatic steatosis (Jin et al., 2013, 2016). Here, we first demonstrated a novel function of RAGE in the regulation of aging‐associated hepatic steatosis. We show that liver‐specific knockdown of RAGE inhibited HFD‐induced hepatic steatosis in old mice, while liver‐specific overexpression of RAGE promoted hepatic steatosis in middle age mice. Therefore, all these data indicate a critical role of RAGE in the regulation of hepatic steatosis. However, a recent study claimed that RAGE‐deficient mice displayed reduced weight gain and visceral fat expansion compared to control mice and RAGE does not play a major role in the development of NASH in a hyperlipidemic mouse model (Bijnen et al., 2018). As RAGE is widely expressed, the whole‐body deletion of RAGE might differ from the liver‐specific suppression of RAGE. Currently, although the reason for this inconsistency is unclear, the animal models and feeding conditions were different from our study. Especially, they did not utilize old mice for comparison. Moreover, consistent with our results, another group found that RAGE KO mice appeared to be protected from the increased liver fibrosis and hepatic steatosis induced by high cholesterol (HFHC) diet with AGEs (Leung et al., 2016). However, the hepatic steatosis status has not been compared between WT and RAGE KO old mice. Thus, further studies are still needed to further investigate the role of RAGE in the liver of old mice using liver‐specific RAGE KO old mice.

To fully uncover the molecular mechanism by which RAGE regulates hepatic steatosis, we showed that PPARα expression was negatively regulated by RAGE. In line with our study, previous research showed that PPARα protein expression was reduced in senescence‐accelerated prone mice 8 (SAMP8) (Angela, Blei, Jaster, & Vollmar, 2011). We profiled the expression levels of the transcription factor PPARα, which is closely associated with fatty acid β‐oxidation in the liver (Li et al., 2014). PPARα is the most important regulator involved in fatty acid β‐oxidation. Previous studies have reported that PPARα gene expression is correlated with disease severity and histological treatment response in patients with non‐alcoholic steatohepatitis (Francque et al., 2015), and several other studies showed that downregulated PPARα induces hepatic steatosis in animal models (Loyer et al., 2016; Lu et al., 2014; Montagner et al., 2016; Zhang et al., 2018), emphasizing its critical role in hepatic steatosis.

Mitochondria play a central role in generating energy from nutrient oxidation. It has been reported that mitochondria play a role in FFA metabolism, and impaired mitochondrial function is thought to contribute to NAFLD (Garcia‐Ruiz, Baulies, Mari, Garcia‐Roves, & Fernandez‐Checa, 2013). In the current study, FFA treatment significantly decreased ATP levels in primary hepatocytes, and RAGE silencing partially rescued FFA‐induced mitochondrial damage and decreased ATP levels. On the other hand, PPARα suppression further decreased ATP levels. It is known that the copy number of mtDNA serves as a parameter for evaluating mitochondrial function (Nassir & Ibdah, 2014). In line with this measure, we found that the RAGE/PPARα axis influenced mtDNA copy number, indicating that the RAGE/PPARα axis is closely associated with mitochondrial dysfunction in primary hepatocytes.

A recent paper showed that RAGE inhibition suppresses PKA signaling, which in turn improves mitochondrial fat oxidation (Hurtado Del Pozo et al., 2019). However, Ching‐Chyuan Hsieh et al showed that the PKA‐p38 signaling was only mildly activated in aging mice liver (Hsieh & Papaconstantinou, 2004, 2006), suggesting that its contribution to hepatic steatosis in aging mice liver is limited. Furthermore, we treated old WT mice contained high levels of TG and steatosis with shRNA‐RAGE and found that the steatosis was reduced; however, phosphorylated CREB, a downstream marker of the PKA signaling was not significantly altered, suggesting that PKA signaling was not significantly altered (Figure S1F). Thus, although we could not completely rule out the possibility that the PKA signaling inactivation participates in the protective effects of RAGE deficiency on the liver of aging mice, the role of this signaling is limited.

Bijnen et al. (2018) claimed that RAGE‐deficient mice displayed reduced weight gain and visceral fat expansion compared to control mice and RAGE does not play a major role in the development of NASH in a hyperlipidemic mouse model. Currently, although the reason for this inconsistency is unclear, the animal models and feeding conditions were different in the two studies. The animal model they utilized are RAGE‐deficient mice and wild‐type littermates, both on Ldlr−/− background. Ldlr knockout mice usually developed severe disorders in cholesterol homeostasis, which may compromise the hepatic triglyceride metabolism. Furthermore, whole‐body RAGE knockout mice were used in their study, while we specifically overexpressing or silencing RAGE in liver. Considering that RAGE is functionally expressed in other metabolic tissues, such adipose tissue, muscle, and islet, our liver‐specific overexpression or knockdown might be better to understand its specific metabolic roles in the liver. The conflicting data between whole‐body knockouts and tissue‐specific knockouts have been reported in many other studies. For example, severe insulin resistance was only observed in liver‐specific insulin receptor (IR) knockout mice and not in skeletal muscle‐ or fat‐specific IR knockout mice (Bluher et al., 2002; Bruning et al., 1998; Michael et al., 2000). Besides, TRB3 was shown to inhibit insulin signaling and promote insulin resistance in the liver (Du, Herzig, Kulkarni, & Montminy, 2003; Koo et al., 2004; Yu et al., 2015). However, serum glucose or insulin levels; insulin sensitivity or glucose tolerance; or energy metabolism were not altered in genetic TRB3‐deficient mice (Okamoto et al., 2007). Therefore, global TRB3 knockout mice displayed normal hepatic insulin signaling and glucose homeostasis (Okamoto et al., 2007). Thus, all these results suggest that mice with liver‐specific knockout/knockdown/overexpression could be a better model to explore the role of target gene in the liver.

HMGB1 is a damage‐associated molecular pattern (DAMP) molecule that triggers the progression of hepatic steatosis by inducing signals upon interaction with RAGE (Anggayasti et al., 2020). We found that HMGB1 expression was also increased in the livers of the old mice, suggesting the aberrant activation of the HMGB1/ RAGE signaling in the livers of the old mice. HMGB1 modulates gene expression in the nucleus, but certain immune cells secrete HMGB1 as an extracellular alarmin to signal tissue damage (Davalos et al., 2013). We speculate that old mice with a large number of senescent liver cells might secrete HMGB1 which activates the RAGE/PPARα axis to promote hepatic steatosis. Hepatic steatosis may progress to hepatocyte injury and the initiation of inflammation, and inflammatory cells such as infiltrating macrophages, T lymphocytes, neutrophils, and DCs all contribute to liver inflammation. We found that liver‐specific overexpression of RAGE caused significant macrophage infiltration and expression of pro‐inflammatory cytokines in the liver of middle age mice, while liver‐specific knockdown of RAGE decreased macrophage infiltration and expression of pro‐inflammatory cytokines in the liver of old mice, suggesting that macrophage infiltration and sequential pro‐inflammatory cytokine expression are required for RAGE's effects on old mice hepatic steatosis. Thus, our data may establish a correlation between senescence burden and non‐cell autonomous sensing of inflammation in the liver.

In conclusion, our present study revealed for the first time that increased RAGE expression was responsible for hepatic TG accumulation in aging mice. We found that RAGE inhibition increased PPARα expression, which further enhanced mitochondrial β‐oxidation (Figure 6e). Thus, the RAGE/PPARα regulatory axis might be a promising therapeutic target for aging‐related fatty liver disease.

## 4 EXPERIMENTAL PROCEDURES

### 4.1 Mouse experiments and human liver tissues

Male C57BL/6 mice aged 8–10 weeks were purchased from the Shanghai Laboratory Animal Company (SLAC, Shanghai). The mice were housed at 20–24°C with a light/dark cycle of 12 hours. Old mice were fed either a high‐fat diet (HFD) or a standard chow diet as described previously (Lu et al., 2014) for 17 months since the mice were 3 months old, while middle‐aged mice were fed a standard chow diet for 10 months. The animal protocol was approved by the Animal Care Committee of Shanghai Jiao Tong University. To collect human liver samples, liver tissues were collected from male patients who underwent regular hepatectomy for hepatic hemangiomas at the Xinhua Hospital affiliated with Shanghai Jiao Tong University from 2016 to 2018. Patients aged 20–50 years were enrolled in the young and middle‐aged group, and patients aged 65–80 years were enrolled in the old group. Data from patients with diabetes, cardiovascular diseases, cerebral infarction history, or a BMI over the range of 19–23 kg/m^2^ were excluded. All patients signed a written consent form. The human study was approved by the Ethics Committee of Xinhua Hospital affiliated with Shanghai Jiao Tong University. The patients’ clinical characteristics are listed in Table S1.

### 4.2 Recombinant adenoviruses and primary hepatocyte isolation, cell culture, and treatments

Recombinant adenoviruses were customized by GeneChem company. RAGE shRNA viruses transcribe RAGE short hairpin RNA to silence the expression of RAGE, while Ad‐RAGE viruses overexpress RAGE. shRNA‐EGFP and Ad‐GFP served as their controls. Viruses diluted in PBS were administered at a dose of 1 × 10^7^ plaque‐forming units (PFU) per well in 12‐well plates or 5 × 10^6^ PFU per well in 24‐well plates. For the animal study, 1 × 10^9^ PFU were injected into middle‐aged wild‐type (WT) mice, or 2 × 10^9^ PFU were injected into old age HFD‐fed mice via the tail vein (Xiao et al., 2014). The methods of isolation and culture of the mouse primary hepatocytes were described previously (Wang et al., 2009).

### 4.3 Cellular and hepatic TG measurements

Cultured cells were harvested by a cell scraper and homogenized by sonication. For lipid determinations, homogenates from cells or liver tissues were extracted with NP40. After evaporation of the organic solvent, the triglyceride content of each sample was measured with the triglyceride measurement reagent (BioVision), according to the manufacturer's instructions.

### 4.4 Glucose and insulin tolerance tests

After 16 hours of fasting, d‐glucose (Sigma) was injected intraperitoneally with 2.0 mg/g body weight for glucose tolerance test. For insulin resistance test, mice were injected with regular human insulin at a dose of 0.75 units/kg of body weight after 6 hours of fasting (Lilly). Blood glucose was measured using a portable blood glucose meter (LifeScan; Johnson & Johnson).

### 4.5 Plasmids and luciferase assays

The PPARα luciferase reporter was customized by the GeneChem Company (Shanghai, China). Human embryonic kidney cells (HEK293T cells) were plated onto 24‐well plates and cotransfected with the 100‐nM pGL3 plasmid construct and Ad‐RAGE or the corresponding control using Lipofectamine 2000 (Invitrogen). Forty‐eight hours post‐transfection, luciferase activity was measured using dual‐luciferase reporter assay system (Promega).

### 4.6 Mitochondrial DNA content quantification by real‐time PCR

To quantify the mitochondrial DNA (mtDNA) content, total genomic DNA was extracted from primary hepatocytes using a NucleoSpin kit (Macherey‐Nagel), and real‐time PCR was performed to detect two mtDNA‐specific sequences, *cytochrome b (MT*‐*CYB)* and 12s rRNA, with 18S used as the nuclear DNA control. The primer sequences are listed in Table S2.

### 4.7 RAGE, PPARα, and HMGB1 silencing and AGE inhibition

Control siRNA and siRNA oligonucleotides targeting RAGE, PPARα, and HMGB1 were synthesized by GenePharma Company. The RAGE siRNA sequences were 5′‐GGUGUAUGCUAGAGCUAUACC‐3′ and 5′‐UAUAGCUCUAGCAUACACCAC‐3′. The PPARα siRNA sequences were 5′‐GCUAGUGUCCGAUAGACAAAG‐3′ and 5′‐UUGUCUAUCGGACACUAGCGG‐3′. The HMGB1 siRNA sequences were 5′‐UCUCUUAUCCAACUAUCACGA‐3′ and 5′‐GUGAUAGUUGGAUAAGAGAUA‐3′. Primary hepatocytes were transfected with 10 nM RAGE, PPARα, and HMGB1 siRNA or non‐silencing control siRNA with RNAiMAX Lipofectamine reagent (Invitrogen). Aminoguanidine (AG) (Sigma‐Aldrich), an AGE inhibitor, was used in primary hepatocytes for AGE inhibition.

### 4.8 Detection of cellular ATP levels and oxygen consumption measurements

Cellular ATP levels were measured with a firefly luciferase‐based ATP assay kit (Beyotime). Briefly, primary hepatocytes, in triplicate sets, were lysed and centrifuged at 12,000 × g for 5 min, and 100 μl of supernatant was mixed with 100 μl of ATP detection solution. Total ATP levels are expressed as nmol/mg protein. The intact cellular oxygen consumption rate (OCR) was measured using a Seahorse XF‐96 extracellular flux analyzer (Seahorse Bioscience) as described previously (Gaude et al., 2018; Xie et al., 2016). The results were obtained by performing in triplicate in 24‐well plates, 4 × 10^4^ hepatocytes each, the protein concentration in each well was measured by BCA assay according to the manufacturer's instructions (Thermo), and the protein concentrations are around 0.2–0.3 μg/ml. The OCR value was normalized to the total protein level in each well. Hepatocytes were treated with DMEM/F12 containing 1 mM BSA‐conjugated oleate acid/palmitate acid (OA/PA) (Sigma‐Aldrich) and incubated for 24 hours before the measurement.

### 4.9 Statistical analysis

The Kolmogorov–Smirnov test was used to analyze the normal distribution of the data. Data are shown as the mean ± *SD*. A 2‐tailed unpaired Student's *t* test was performed to compare between two groups. 1‐way ANOVA followed by the Bonferroni test was used to compare more than two groups. Statistical significances were displayed as *p < 0.05, **p < 0.01, ***p < 0.001, ^###^ *p* < 0.001.

## CONFLICT OF INTEREST

No potential conflict of interest relevant to this article was reported.

## AUTHOR'S CONTRIBUTIONS

J.W. and H.C. gathered the data, X.W. obtained clinical materials, X.X, X.S., S.C., and X.L. provided technical assistance for the study, J.J., Q.S., D.C., and B.Liu provided critical comments on the manuscript, B.L., directed the project, reviewed, and wrote the manuscript.

## Supporting information

Appendix S1

## Data Availability

The data that support the findings of this study are available from the corresponding author upon reasonable request.
