# Supplementary material for: Aging‐induced aberrant RAGE/PPARα axis promotes hepatic steatosis via dysfunctional mitochondrial β oxidation
Source: Aging Cell. 2020 Sep 16;19(10):e13238. doi: 10.1111/acel.13238 (PMC7576254; doi:10.1111/acel.13238)
Supplement: Supplementary file 1 — Appendix S1 [file ACEL-19-e13238-s001.docx]

**Supplementary Figure 1**

**
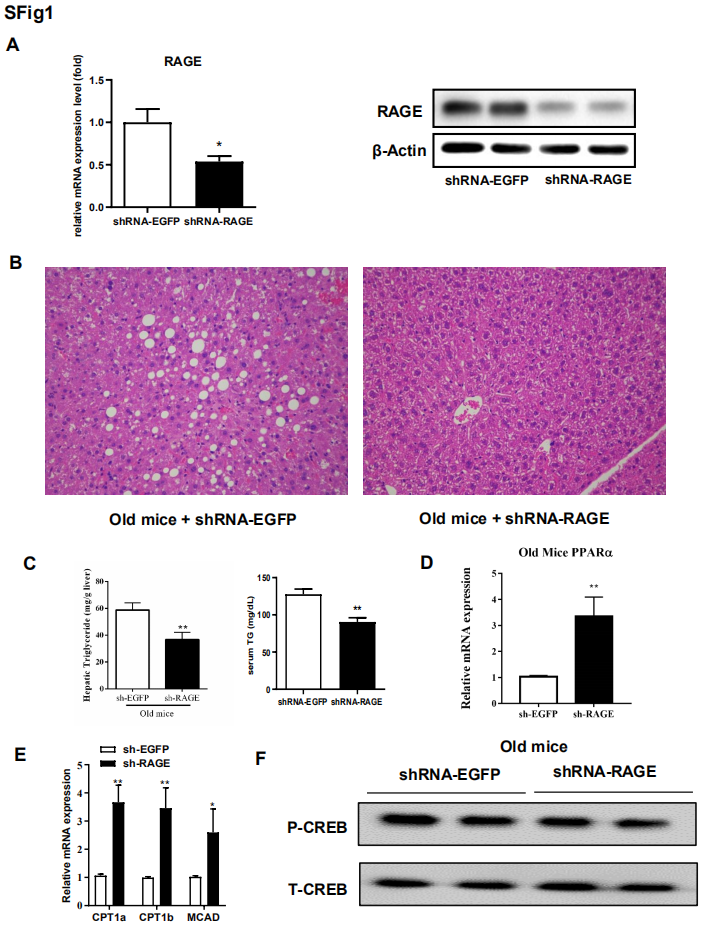
**

**Supplementary Figure 2**

**
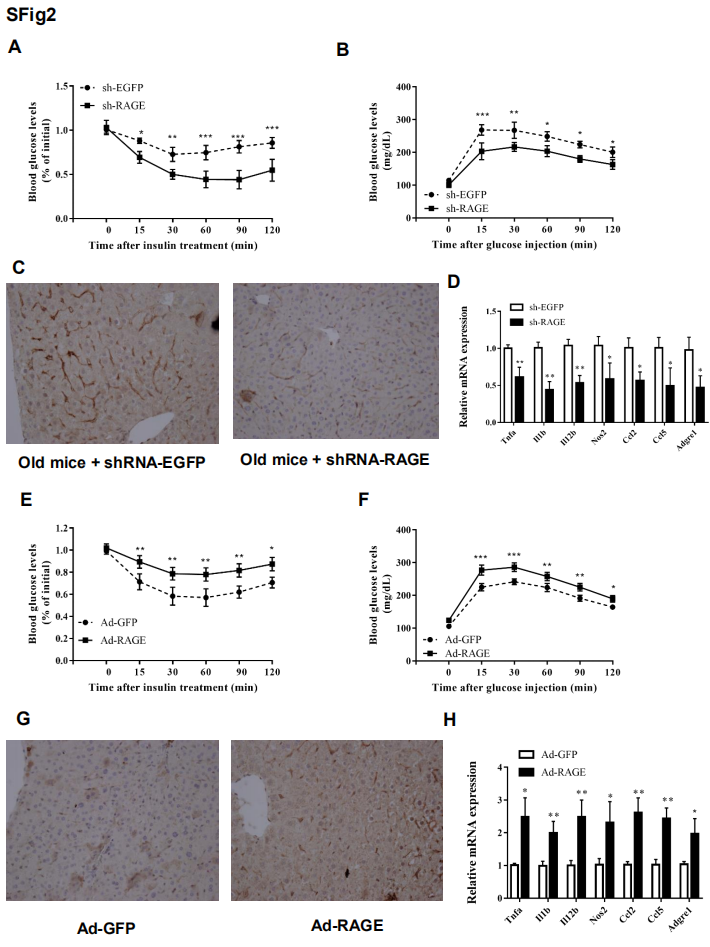
**

**Supplementary Figure 3**

**
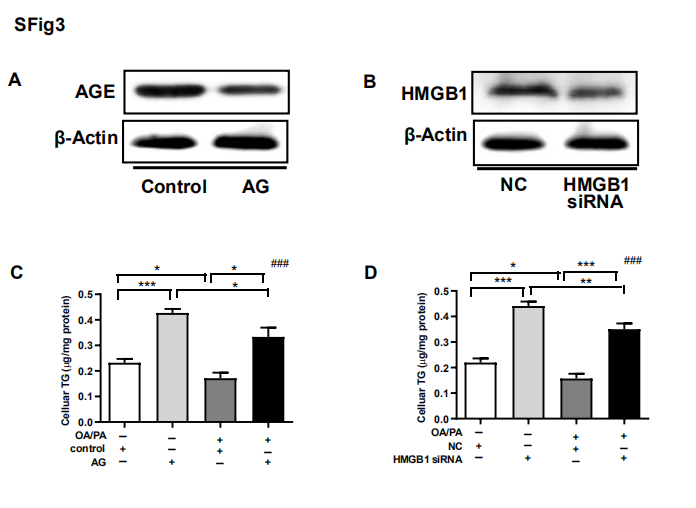
**

**Supplementary Figure 4**

**
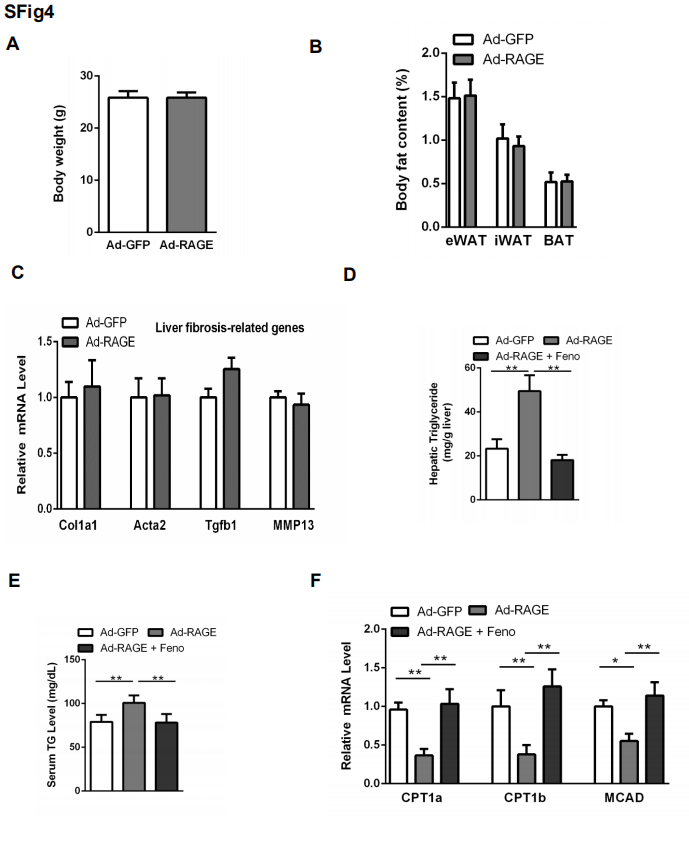
**

**Supplementary Table 1. Clinical characteristics in Young & Middle and Old patients**

|  |  |  | **Young&Middle(n=8)** | | **Old(n=8)** |  | ***P* value** |
| --- | --- | --- | --- | --- | --- | --- | --- |
| Age,y |  |  | 37.6±7.2 | | 72.8±4.8 | | 0.305 |
| BMI,kg/m^2^ | |  | 21.5±1.2 | | 21.4±1.0 | | 0.536 |
| Waist circumference,cm | | | 78.5±2.5 | | 81.3±2.8 | | 0.767 |
| Hip circumference,cm | | | 89.9±4.6 | | 88.6±2.5 | | 0.138 |
| Waist to hip ratio | |  | 0.87±0.04 | | 0.92±0.04 | | 0.742 |
| SBP,mmHg | |  | 114.8±13.0 | | 120.6±16.1 | | 0.549 |
| DBP,mmHg | |  | 67.1±9.8 | | 72.6±13.2 | | 0.446 |
| Lipids |  |  |  |  |  |  |  |
| TC,mmol/L | |  | 3.33±0.59 | | 3.39±0.51 | | 0.713 |
| TG,mmol/L | |  | 0.98±0.26 | | 1.16±0.46 | | 0.162 |
| LDL-c,mmol/L | |  | 2.70±0.76 | | 2.38±0.70 | | 0.846 |
| HDL-c,mmol/L | |  | 1.55±0.41 | | 1.44±0.37 | | 0.789 |
| Fasting plasma glucose,mg/dL | | | 89.78±13,99 | | 88.65±16.03 | | 0.728 |
| HbA1c,% |  |  | 4.95±0.51 | | 5.03±0.56 | | 0.802 |
| Uric acid,mmol/L | |  | 289.00±54.15 | | 310.50±51.55 | | 0.364 |

**Supplementary Table 2. Primer sequences**

| Genes | Forword primer(5' -> 3') |  | Reverse primer(5' -> 3') |  |
| --- | --- | --- | --- | --- |
| RAGE | CGGATTGGCGAGCCACTGGT |  | CTGTCCGGCCTGTGTTCAGTTTC | |
| PPARα | ACCACAGTAGCTTGGAGCTCGG | | GGGCAGAGTGGGCTTTCCGT |  |
| CPT1a | AGGGGGCTTTGGACCGGTTG |  | TCAGGTGCCTTCCAAAGCGATGA | |
| CPT1b | CCACCGCGGAAGGTGCCGA |  | TACACGCCCCTGAGGATGCCA | |
| MCAD | CAGAGGAGTCCCGCGTTCGG |  | CGAACCCCGCTGCCATGTTG |  |
| cytochrome b | CTTGGCGCCTGCCTGATCCT |  | GGCCTCGCCCGATGTGTAGG |  |
| 12s rRNA | TGCAAGCATCCCCGTTCCAGT |  | GAGCTGCATTGCTGCGTGCT |  |
| β-Actin | CATGTACGTTGCTATCCAGGC |  | CTCCTTAATGTCACGCACGAT |  |
| RAGE* | GGGTCACAGAAACCGGCGATG | | GCCACAGGATAGCCCCGACG |  |
| PPARα* | CGCAGCTGTTTTGGGGGCTG |  | AACTTGGCTCTCCTCTAAGTTCCCC | |
| CPT1a* | CTCCGCCTGAGCCATGAAG |  | CACCAGTGATGATGCCATTCT |  |
| CPT1b* | TCTTCTTCCGACAAACCCTGA |  | GAGACGGACACAGATAGCCC |  |
| MCAD* | AACACAACACTCGAAAGCGG |  | TTCTGCTGTTCCGTCAACTCA |  |
| Col1a1* | GCTCCTCTTAGGGGCCACT | | CCACGTCTCACCATTGGGG |  |
| Acta2* | GTCCCAGACATCAGGGAGTAA | | TCGGATACTTCAGCGTCAGGA |  |
| TGFB1* | CTCCCGTGGCTTCTAGTGC |  | GCCTTAGTTTGGACAGGATCTG | |
| MMP13* | CTTCTTCTTGTTGAGCTGGACTC | | CTGTGGAGGTCACTGTAGACT |  |
| Tnf* | AGCCCCCAGTCTGTATCCTT |  | CTCCCTTTGCAGAACTCAGG |  |
| Il1b* | TGGCAACTGTTCCTGAACTCAA | | AGCAGCCCTTCATCTTTTGG |  |
| Il12b* | CCAGAGACATGGAGTCATAG |  | AGATGTGAGTGGCTCAGAGT |  |
| Nos2* | GAGGCCCAGGAGGAGAGAGATCCG | | TCCATGCAGACAACCTTGGTGTTG | |
| Ccl2* | AGGTCCCTGTCATGCTTCTG |  | TCTGGACCCATTCCTTCTTG |  |
| Ccl5* | TGCCCACGTCAAGGAGTATTT |  | TTCTCTGGGTTGGCACACACT |  |
| Adgre1* | ATCCTTGGCCATCCGGCAGA |  | GCAAAGCCAGGGTGGCAAGT |  |
| β-Actin* | GGCTGTATTCCCCTCCATCG |  | CCAGTTGGTAACAATGCCATGT | |
| cytochrome b* | AGTGCGTGTTGCTCGACAA |  | GCGGTGTGCAGTGCTATCAT |  |
| 12s rRNA* | CCACCGCGGTCATACGATTA |  | TTGGGTCTTAGCTGTCGTGT |  |
| 18S | TAGAGGGACAAGTGGCGTTC |  | CGCTGAGCCAGTCAGTGT |  |

*mouse targets

**Supplementary Figure Legends**

Supplementary Figure 1. **Effect of shRNA-RAGE on hepatic steatosis in old mice.** Old C57BL/6 mice were injected with shRNA-EGFP or shRNA-RAGE virus, and RAGE-knockdown efficiency was determined by real-time PCR (A). In addition, assays for western blotting (A), H&E staining of liver sections (B), and hepatic and serum TG levels (C) were performed, PPARα mRNA expression was determined by real-time PCR (D), the expression levels of *CPT1a*, *CPT1b* and *MCAD* were measured with real-time PCR (E), phosphorylated and total CREB protein expression were determined by western blot (F).

Supplementary Figure 2. Glucose and insulin tolerance tests of aged C57BL/6 mice injected with shRNA-EGFP and shRNA-RAGE (A-B). Macrophages staining and expression of hepatic pro-inflammatory cytokines in aged C57BL/6 mice injected with shRNA-EGFP and shRNA-RAGE (C-D). Glucose and insulin tolerance tests of middle-aged C57BL/6 mice injected with Ad-GFP and Ad-RAGE (E-F). Macrophages staining and expression of hepatic pro-inflammatory cytokines in middle-aged C57BL/6 mice injected with Ad-GFP and Ad-RAGE (G-H).

Supplementary Figure 3. (A)AGE efficiency was determined by western blotting analysis in primary hepatocytes treated with AG or control. (B)HMGB1 efficiency was determined by western blotting analysis in primary hepatocytes transfected with HMGB1 siRNA or NC siRNA. (C)After primary hepatocytes were treated with AG or control and stimulated with OA/PA or BSA control, cellular TG were examined. (D)After primary hepatocytes were transfected with HMGB1 siRNA or NC siRNA and stimulated with OA/PA or BSA control, cellular TG were examined.

Supplementary Figure 4. C57BL/6 mice injected with Ad-GFP and Ad-RAGE (A-C), body weights (A) and fat pad masses (B) were measured, the expression levels of Col1a1,Acta2,Tgfb1 and Mmp13 were measured with real-time PCR (C). C57BL/6 mice injected with Ad-GFP and Ad-RAGE with or without Feno (D-F), hepatic and serum TG levels (D-E) were performed, the expression levels of CPT1a, CPT1b and MCAD were measured with real-time PCR (F).

**Supplementary Experimental Procedures**

***RNA isolation and quantitative real-time PCR***

Total RNA and protein extracts were isolated from hepatocyte lysates or livers for the gene expression analysis. The primer sequences are listed in Supplementary Table 2. Total RNA was extracted from the liver tissues and cell lysates with TRIzol according to the manufacturer’s instructions (Invitrogen, USA). Total RNA (1 µg) was reverse transcribed into cDNA using oligo-dT primers (Promega, USA). Real-time PCR analysis was carried out using SYBR Green Premix Ex Taq (TaKaRa, Otsu, Japan) on a Light Cycler 480 (Roche, Basel, Switzerland) with β*-actin* serving as an internal control.

***Western blotting and antibodies***

Homogenized tissues and cells were lysed in RIPA buffer containing 1 × PBS, 1% NP40, 5mM EDTA, 0.1% sodium dodecyl sulfate (SDS), 1mM Na_3_VO_4_, 1% phenylmethanesulfonylfluoride, complete protease inhibitor cocktail (Sigma) and complete phosphatase inhibitors. The lysates were centrifuged at 12,000 g for 10 min at 4°C to remove the insoluble materials, and the supernatants were boiled in SDS loading buffer. The boiled samples were separated by 10% SDS–polyacrylamide gel and electroblotted to nitrocellulose transfer membranes (Whatman, GE Healthcare). The membranes were blocked with 5% milk and incubated with different antibodies, followed by incubation with secondary antibodies. Primary antibodies used in Western blotting included anti-RAGE (Abcam, IL, USA), anti-PPARα (Millipore, MA, USA), anti-β-actin (Cell Signaling, MA, USA), anti-HMGB1, anti-AGE (Abcam, IL, USA), anti-p-CREB (Cell Signaling, MA, USA) and anti-t-CREB (Cell Signaling, MA, USA).

***Histopathologic analysis***

Liver tissues were fixed in 10% neutral buffered formalin and embedded in paraffining. Sections were subjected to standard hematoxylin and eosin staining. For oil red O staining, liver tissues were fixed in 4% paraformaldehyde in PBS, embedded in optimum cutting temperature compound (OCT), and cryosectioned. Frozen liver sections were stained with 0.15% oil red O according to standard procedures. For immunofluorescence microscopy, cryosections of the livers were fixed with 10% formalin, blocked with 5% BSA (Sigma-Aldrich), and labeled with the antibody against mouse F4/80 (Abcam, ab16911) for 3 hours, followed by incubation with a fluorophore-conjugated secondary antibody (Thermo Fisher Scientific) for 1 hour according to standard procedures.
